# Diabetic retinopathy detection using Bilayered Neural Network classification model with resubstitution validation

**DOI:** 10.1016/j.mex.2024.102705

**Published:** 2024-04-06

**Authors:** Herman Khalid Omer

**Affiliations:** Department of Information Technology, Technical College of Duhok, Duhok Polytechnic University, Duhok, Kurdistan Region-IRAQ

**Keywords:** Diabetic retinopathy (DR), Feature extraction, Machine learning, Resubstitution validation, Diabetic Retinopathy Detection Using Bilayered Neural Network

## Abstract

In recent years, eye diseases in diabetic patients are one of the most common has been diabetic retinopathy (DR). which leads to complete blindness in advanced stages. Diabetes affects the blood vessels in the retina and causes vision loss. One of the ways to decrease the risk of this issue is to detect diabetic retinopathy in its early stages. This study describes a computer-aided screening system (DREAM) that uses a neural network classification model in machine learning to assess fundus images with different illumination and fields of vision and provide a severity grade for diabetic retinopathy. Moreover, the methodology of this study based on:

• Enhancement techniques have been used on dataset images, histogram equalization, noise reduction and image scaling,
• vSLAM has been selected as feature extraction,
• Bilayered Neural Network under resubstitution validation used as a classification model.

Finally, after testing on the DR severity grading system is tested on 6332 images of detection of diabetic retinopathy images, the result of the ROC curve is 0.985 on image dataset and obtains accuracy reached 98.5%. The classification result has been founded under MATLAB platform, beside it that be work with real time analysis and detection when patient eyes analysis.

## Graphical abstract

**Figure:**
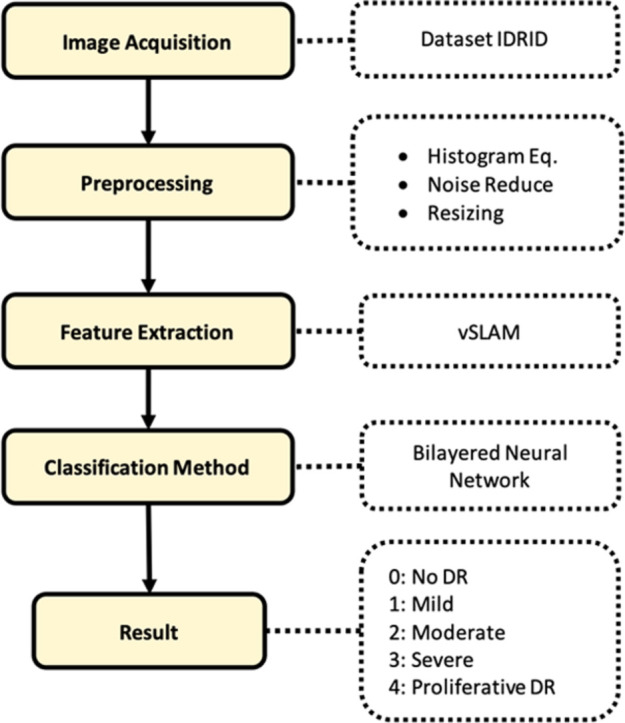

Specifications Table

**Table:** 

Subject area:	Computer Science
More specific subject area:	Image processing
Name of your method:	Diabetic Retinopathy Detection Using Bilayered Neural Network
Name and reference of original method:	N/A
Resource availability:	Indian Diabetic Retinopathy Image Dataset (IDRID)

**Method details**

## Introduction

One of the most serious illnesses in the modern world is diabetic retinopathy (DR). Consequently, a number of human eyes suffer from substantial impairments to their eyesight. The World Diabetes Federation reports that over 463 million people worldwide suffered from diabetes in 2019. In addition, it is predicted that by 2045, there would be 700 million people with the disease worldwide. As a result, the number of diabetes patients is rising quickly in this regard. Diabetic retinopathy, which causes visual loss, is also rising steadily.

Currently, DR is a more complex condition that damages the retina's blood vessels and raises blood sugar levels. Early intervention with appropriate DR medication can reduce the chance of blindness. These days, it's critical to identify and diagnose DR. Globally, there is a challenge and progress in DR identification to treat DR at an earlier stage of the illness.

Using emerging technology, especially machine learning and deep learning, is one of the most often used strategies for improving performance in this industry. Many scientists are trying to figure out how to find the DR at an early stage of their study. Early disease detection and prevention were suggested by the DR literature assessment. The ending of this paper is arranged as; proposed system method for detection is represented in methodology part. Results and discussions are represented in Results and discussion part, conclusion of proposed work is explained in final part of diabetic retinopathy is made possible by the prompt identification. In relation to the dataset, feature extraction, and classifier, some of the literature review is revealed in this study. Furthermore, several classifiers and datasets were described in order to categorize diabetic retinopathy into distinct groups.

Rajkumar et al. [1], developed a transfer learning technique to identify normal and abnormal images from the 35,000 images in the Kaggle dataset using a CNN architecture based ResNet-50 model. The study of the results showed that the ResNet-50 model had an accuracy of 89.4%. It tested using the Kaggle database, and the results showed 98.43% accuracy, 97% specificity, and 98% sensitivity. Lands and colleagues [2] created and employed a deep learning-based method for classifying DRs, For DR detection, three CNN architectures—ResNet, DenseNet129, and DenseNet169—are utilized. The validation loss for the ResNet classifier was 67%, the DenseNet129 classifier was 32%, and the DenseNet169 classifier was 21%. Lazuardi et al. [3], makes use of the Kaggle dataset and the deep convolutional neural network (DCNN) architecture with the EfficientNet-B4 and EfficientNet-B5 classifiers. The two classifiers' respective accuracy is (i) 83.67% for EfficientNet-B4 and (ii) 83.89% for EfficientNet-B5. Raj et al. [4], created a convolutional neural network to locate characteristics related to microaneurysms and blood vessels. With the use of the Kaggle database, the accuracy was 95.41%. Kamblea and Kokate[5], RBF Neural Network classifier for DR detection is suggested. Using the DIARETDB0 and DIARETDB1 datasets, accuracy of 71.2% for the former and 89.4% for the latter was attained.

Alyoubi et al. [6], proposed a five-stage classification technique for the DR images. The APTOS 2019 dataset's several DR phases are distinguished using YOLOv3 and the CNN512 deep learning-based models. It achieves an accuracy of 89% for YOLOv3 and 88.6% for CNN512. Mishra et al. [7], developed a DenseNet model. To identify the DR phases, the proposed approach uses the APTOS dataset to extract features from fundus images stored in a database. The accuracy of 0.9611 that was obtained is compared to DenseNet121 and VGG16. Lavanya et al. [8], DR was detected by implementing the deep learning architecture. 90% of the 35,126 photos that were extracted from the Kaggle database were used for training, while the remaining 10% were used for validation. The achieved training accuracy is 92%. According to the findings, this approach is helpful for primary screening as it can classify the kind of retinal image. Sinthanayothin et al. [9] carried out research to categorize retinal images as normal or pathological. The radial basis function (RBF) kernel and color characteristics were utilized by the author to train the SVM algorithm. The accuracy displayed by the system is 82.6%. Pradeep et al. [10], proposed system was presented. Retinal images are utilized to extract texture characteristics, which are then applied to create SVM classifier using the RBF kernel. The accuracy rate of the suggested approach in differentiating between proliferative, non-proliferative, and normal DR was 95.7%.

Gulshan et al. [11] provide a method for DR detection. The result of the deep learning technique is combined with manually created features using the SVM with linear kernel. In terms of referable DR detection, the suggested method produced a receiver operating characteristic (ROC) curve of 0.99. Quellec et al. [12], created an SVM-based DR detection method for optical coherence tomography (OCT) and fundus images. Using a Kaggle dataset, this model's receiver operating characteristic curve (AUC) was 0.93. Qian et al. [13], offers a deep learning method for DR identification. These techniques combine datasets from previous Kaggle competitions. Convolutional neural networks (CNNs) based on Res2Net and DenseNet for feature extraction and classification. It obtained an accuracy of 83.2%. Seetah et al. [14], makes use of a convolutional neural network to recognize objects by using fundus images. Training and testing are conducted using the Kaggle dataset. This system does well in increasing its 98.25% specificity, 98% sensitivity, and 84% accuracy. Bilal et al. [15], a novel hybrid approach for DR detection with the Kaggle dataset is proposed. It uses three classifiers: binary trees with voting, K-nearest neighbors, and Support Vector Machines. It reaches its highest accuracy of 98.06%, sensitivity of 83.67%, and specificity of 100%.

Wu and Hu[16], developed a transfer learning-based approach for DR recognition. Training data for this model includes VGG19, InceptionV3, and Resnet50. The complete network was trained using the ImageNet dataset. According to the experimental results, InceptionV3 can classify data with a maximum accuracy of 60%. Kolla and Venugopal[17], suggest using Binary Convolutional Neural Networks for Effective DR Classification (BCNN). Large fundus images can be used to identify the DR using the BCNN classifier. An experiment with a 37% memory usage reduction and a 49% runtime accuracy was carried out using the Kaggle dataset. Maswood et al. [18]. presented a pretrained CNN classifier deep learning model to distinguish five different DR classes. The photos are trained and tested using the Kaggle Dataset. The results show that the accuracy for testing is 0.9333, while the accuracy for training is 0.9402. Qomariah et al. [19], SVM and CNN classifiers are used in the proposed DR classification system. Using 77 and 70 retinal images from the Messidor datasets with bases 12 and 13, respectively, the suggested approach is evaluated. Based on the results, base 12 and base 13 have maximum accuracy rates of 95.83 and 95.24%, respectively. Menaouer et al. [20], suggested a Hybrid DL model with the classifiers VGG16 and VGG19, utilizing the DCNN technique. Using the APTO-S 2019 dataset, the results showed an accuracy of 90.60%, a recall of 95%, and an F1 score of 94%. Yu et al. [21], created an automated image processing method using Support Vector Machine for image classification and multilayer Gabor filtering for vessel segmentation to identify neovascularization in the optic disc area. Using the 424 retinal images from the Kaggle Dataset, the selected features were trained and assessed. It achieves 92.90% sensitivity, 96.30% specificity, and 95.23% accuracy. Raja Sarobin et al. [22], used the Kaggle dataset to develop a CNN model that used the ResNet and DenseNet classifier to identify the DR. With the aid of deep learning architectures, the blood vessel exudates and cotton wool spot characteristics are retrieved and categorized. It achieves 96.22% accuracy on ResNet and 93.18% accuracy on DenseNet. Gao et al. [23], developed a DCNN network approach that achieves an accuracy of 88.72% for grading the severities of DR fundus images from the Kaggle datasets. When the classifier was used in this suggested technique for the DR diagnosis on the cloud computing platform, its consistency rate was 91.8%. Agurto et al. [24], suggested a KNN classifier to locate macula area exudates. The optimal instantaneous amplitude (IA) thresholding is used for the feature extraction process in order to identify the exudate regions. The MESSIDOR dataset was used for training and testing the pimages, and an accuracy of 0.96 and 0.97 was obtained in the receiver operator characteristic curve (AUC). Zubair et al. [25], proposed a Microaneurysm Retinal vein Haemorrhage Exudate (MRHE) and Feature Enhancement and Edge Detection (FEED) as a feature exrtation, Deep Convolutional Neural Network (D-CNN) has been used to DR classification, and the final accuracy result was 95.33%.

### Method workflow

**Fig. 1:**
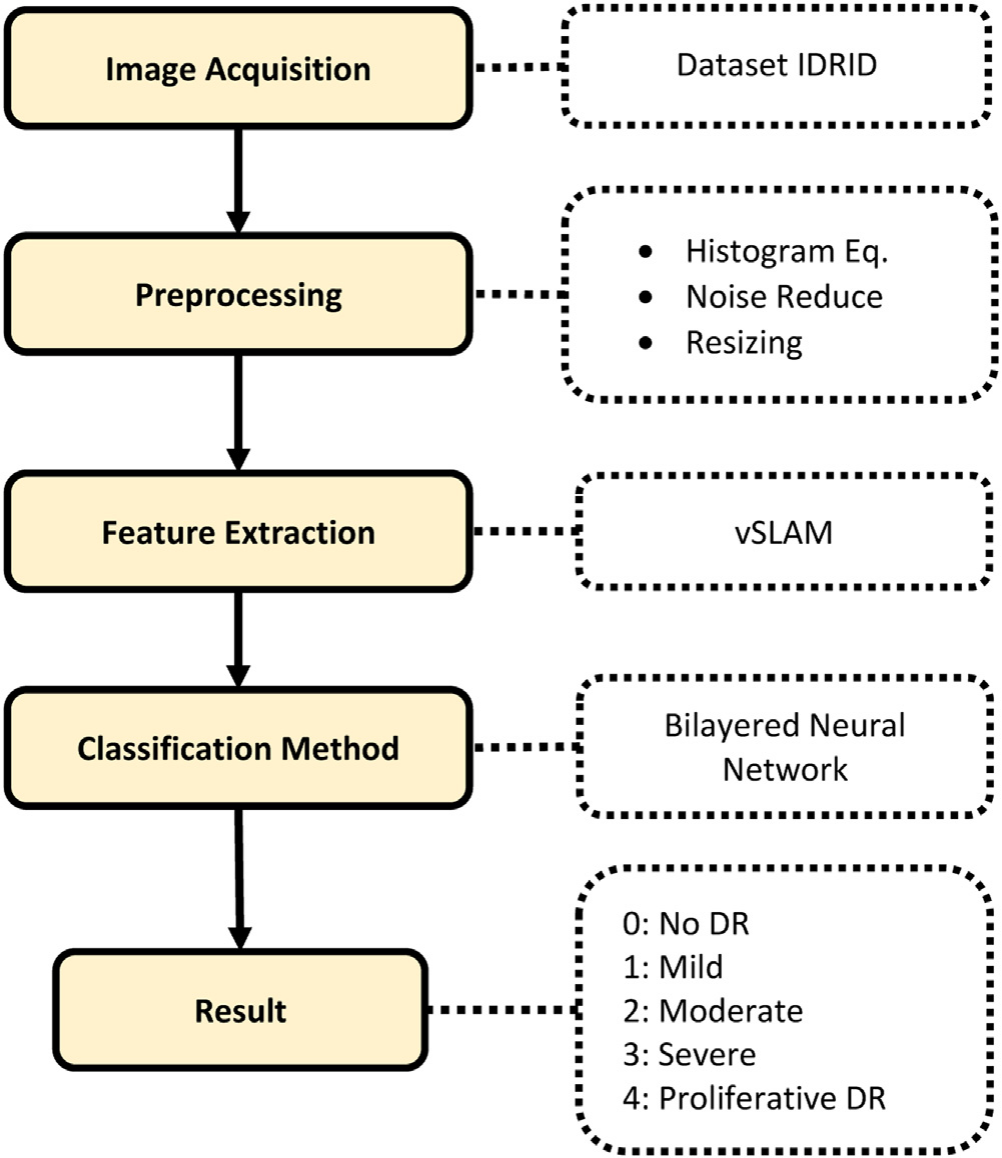
Proposed Block Diagram of the DR Classification.

The Implementation which was done under selected set of steps and algorithms, each step would be clarified in this section, beside that it would be explained functions that used. The paper methodology as the following, as shown in Fig. 1:

### Materials and method validation

#### Image preprocessing

In preprocessing stage, first of all collect image from dataset beside load official diagnostic diabetic retinopathy depend on Indian Diabetic Retinopathy Image Dataset (IDRID) [26] to workspace, however this phase include enhancement for dataset to more detailly which as the following:

#### Histogram equalization

**Fig. 2:**
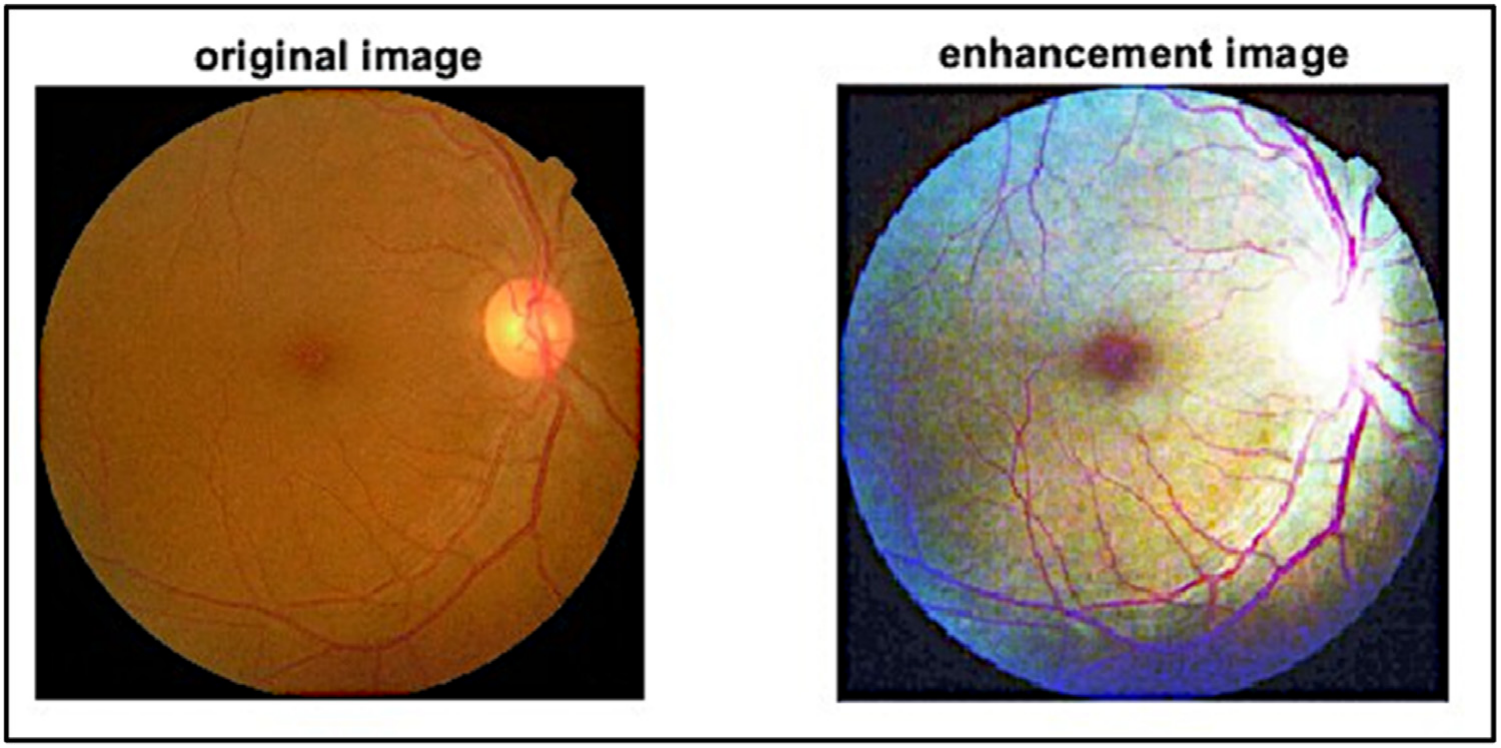
Applied Histogram Equalization.

It is first step in preprocessing phase. Histogram equalization entails changing the intensity levels to roughly match a predefined histogram with the output image's histogram [27]. At the beginning of this step, apply the histogram equalization function to all images in the dataset. In the same time, for the color space red, green, and blue arrays separately, keep images as a color space level to avoid losing color details for the next step, as shown in in Fig. 2.

**Fig. 3:**
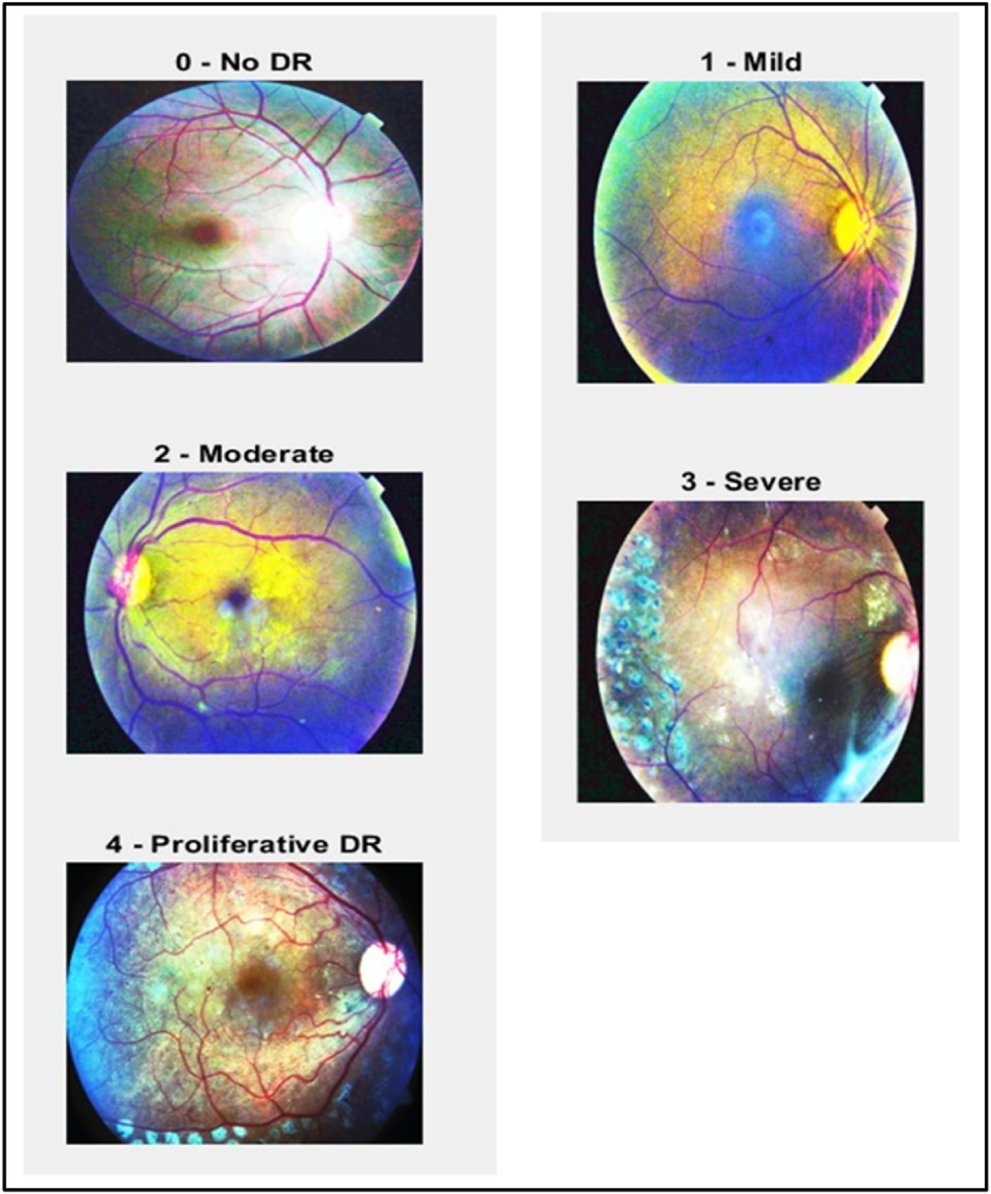
Histogram Equalization Effect for Each Diabetic Retinopathy Stage.

Histogram Equalization normally applied to adjust pixel intensity value, that make it on cover 8-bit range color, and increase contrast of image that be appearance more details. Beside it decrease high contrast and increase low contrast in mid-range contrast, this function help to increase accuracy in the final phase, if looking left image in Fig. 3, the image color space over same range beside it if looking right image, the white spot appeared clearly with colored other part of fundus [28].

#### Noise reducing

**Fig. 4:**
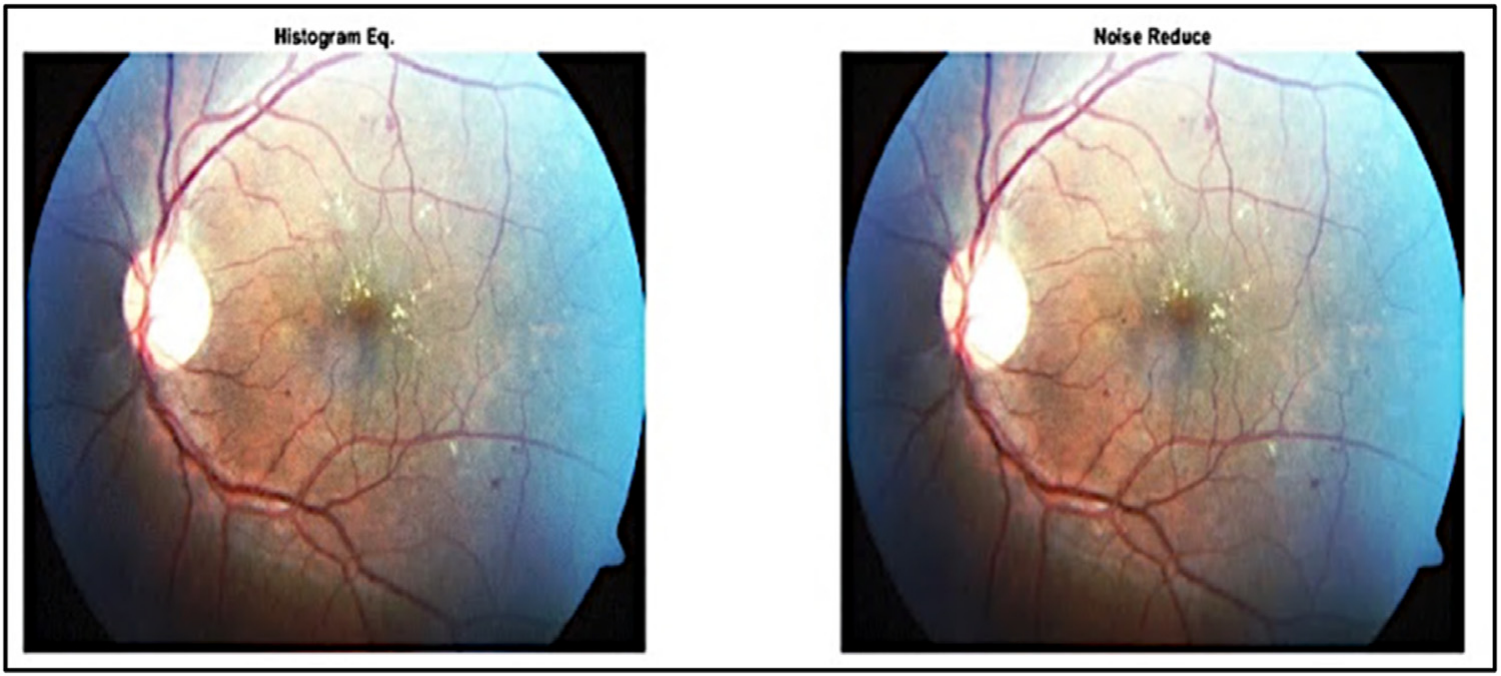
Applied Median Filter for Noise Reduce.

To reduce image from noise and be clear with advantage pixel value, here in this step of the preprocessing Median filtering for noise reducing function has been applied after Histogram Equalization section. In image processing, this nonlinear method is frequently employed to minimize "salt and pepper" noise. When reducing noise at the same time, a median filter works better than a convolutional one. In image processing, this nonlinear method is frequently employed to minimize "salt and pepper" noise [29]. When reducing noise at the same time, a median filter works better than a convolutional one, as shown in Fig. 4.

#### Image resizing

You may resize an image to make it bigger or smaller without removing any parts of it. Resizing a image modifies its proportions, which usually has an impact on the file size and quality of the image. Reduce the size of large image, it is the most common reason to make it easier to extraction features in the same size. The size 200✕200 have been applied which is best size to get highest accuracy and short real time running.

#### Feature extraction

The method of locating and obtaining features from unprocessed data is called feature extraction. It entails dissecting and determining an image's distinctive visual elements, such as its colors, textures, and forms. It makes it possible to retain important information while reducing data needed for analysis more effectively. The four characteristics for DR early detection are described in this research.

A consequence of diabetes called diabetic retinopathy can lead to the development of microaneurysms (MAs), which are tiny outpouchings in the walls of blood vessels in the retina [17]. These tiny aneurysms harm the retina and result in blindness. The location where the optic nerve enters the eye and joins the retina is called the optic disc (OD), sometimes referred to as the blind spot [21]. It is a significant retinal landmark that may be utilized to distinguish between different retinal diseases. Areas of bleeding inside the retina are called hemorrhages (HEs), and they can be brought on by a number of illnesses, including age-related macular degeneration, hypertension, and diabetic retinopathy. The fovea, a tiny central depression in the retina, is in charge of fine-grained vision. It is crucial for the detection and treatment of many retinal diseases. The Ensemble classifier receives the extracted characteristics.

**Fig. 5:**
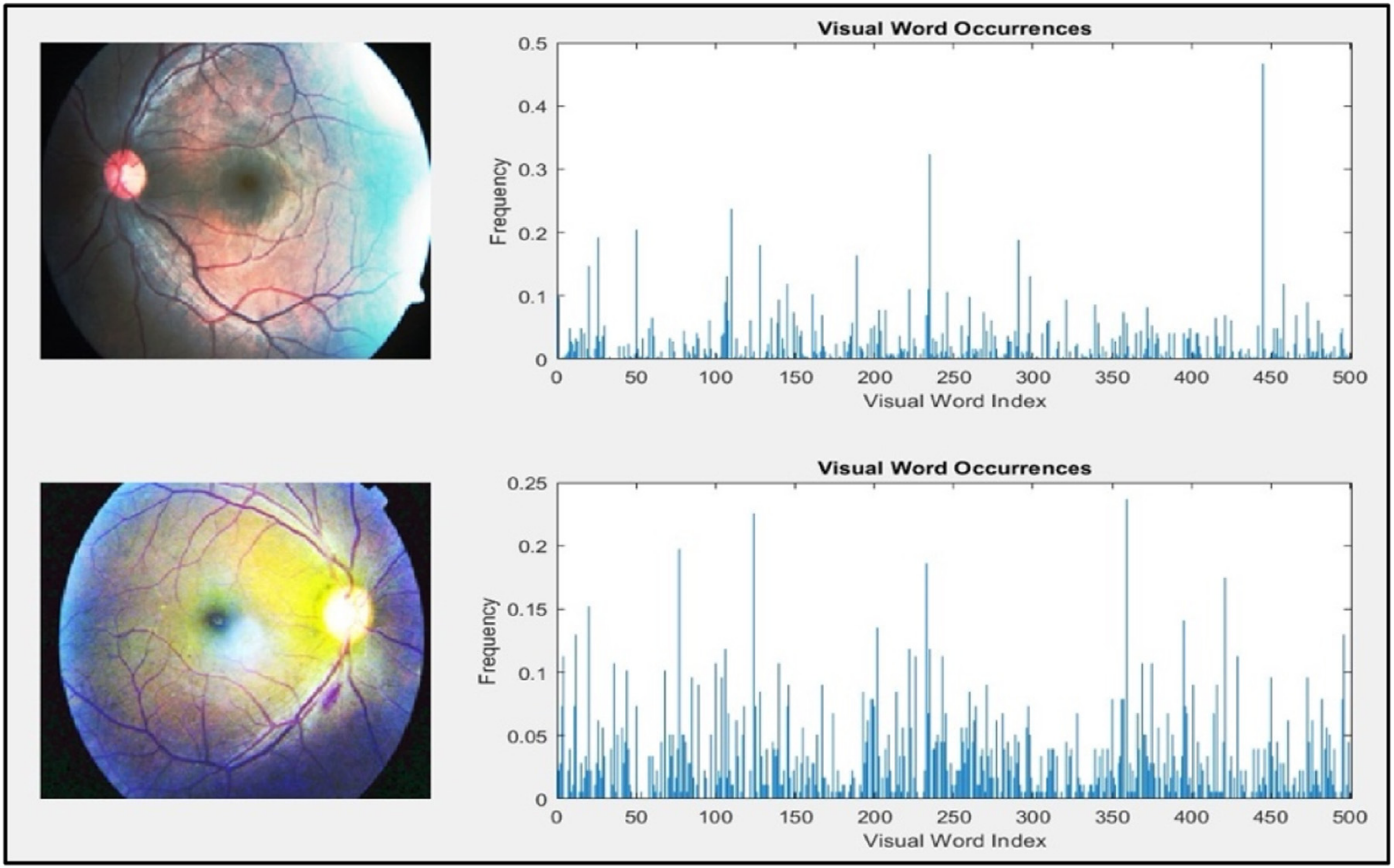
Visual Simultaneous Localization Features.

Visual simultaneous localization and mapping (vSLAM), refers to the method of mapping the environment and determining a detail of image's location and orientation in relation to its surroundings. Visual SLAM literature uses these common terms [30]:

• Key Frames: a portion of the video frames with tracking and localization hints included. Typically, two important frames in a row signify a significant visual shift brought on by a camera movement.
• Map Points: a set of three-dimensional world points that correspond to the environment's map that was pieced together using the key frames.
• Covisibility Graph: a graph where the nodes are key frames. If two key frames share map points, then they are connected by an edge. The quantity of shared map points determines an edge's weight.
• Recognition Database: a database that, using the input bag of characteristics, records the visual word-to-image mapping. Find out if a location has ever been visited by looking through the database for an image that resembles the query image visually, as shown in Fig. 5.

#### Neural network classifiers model

Although neural network models may be used for multiclass classification and generally have decent predicted accuracy, they can be difficult to comprehend. The size and quantity of fully linked layers in the neural network boost model adaptability [31]. As shown in Table 1.

**Table 1:** Neural Network Classifier Types.

Type of Classifier	Flexible Model	
Narrow Neural Network	Medium	rises with the First layer size setting
Medium Neural Network		
Wide Neural Network		
Bilayered Neural Network	High	rises with the First- and Second-layer size settings
Trilayered Neural Network		rises with the First-, Second-, and Third-layer size settings

For classification, each model is a fully connected, feedforward neural network. Each succeeding layer of the neural network has a link from the layer before it, with the first completely connected layer having a connection from the network input (predictor data). Every completely linked layer adds a bias vector after multiplying the input by a weight matrix. Every completely linked layer is followed by an activation function. The output of the network is predicted labels and classification scores (posterior probabilities), which are generated by the last fully connected layer and the softmax activation function that follows. for further details.

#### Neural network model hyperparameter options

**Fig. 6:**
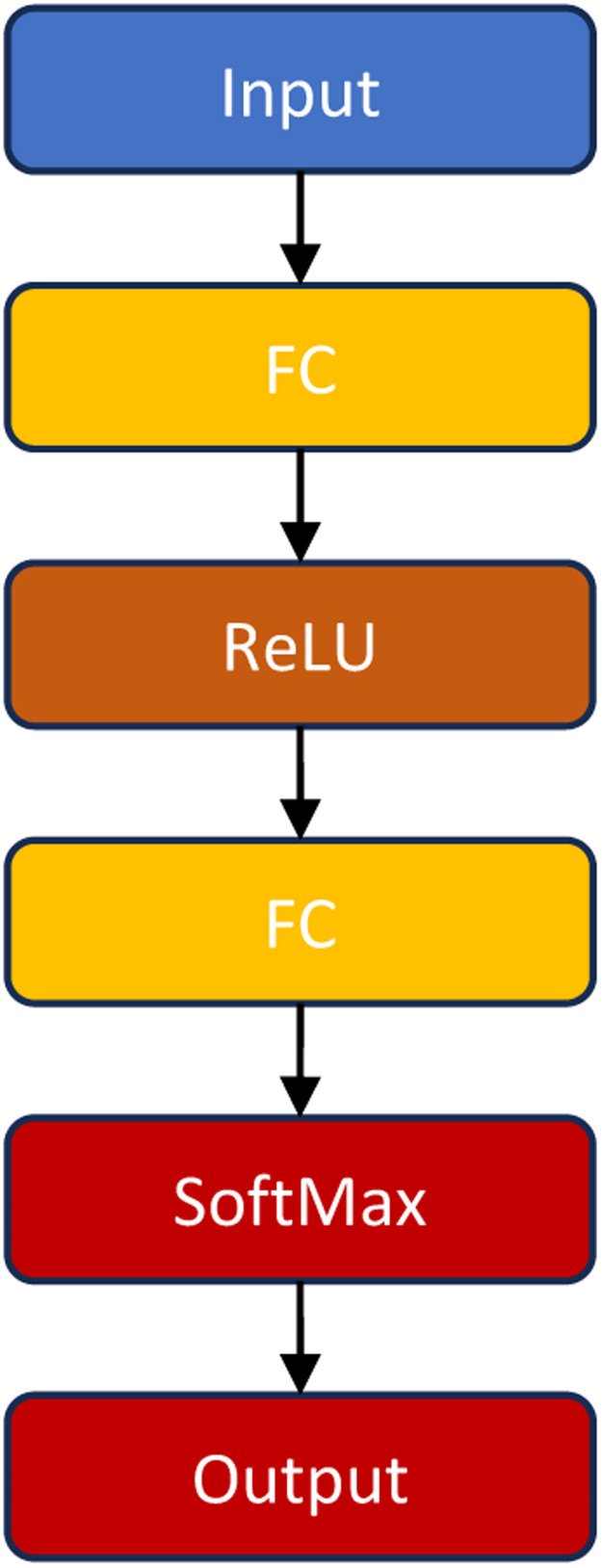
Structure of Neural Network Classifiers.

Classification Learner in Neural network classifiers use the fitcnet function include these following selections [32], and Structure of Neural Network Classifiers is clarified in Fig. 6.

Number of fully connected layers: Provide the neural network's total number of completely connected layers (do not include the last fully connected layer for classification). A maximum of three completely linked levels are available.

First-, second- and Third-layer size: Provide the dimensions of every completely linked layer—not the last fully connected layer—that you have identified. If you decide to build more than one fully connected layer in your neural network, you might want to define layers with progressively smaller sizes.

Activation: With the exception of the last fully linked layer, specify the activation function for each fully connected layer. Softmax is always the activation function for the final completely linked layer. ReLU, Tanh, None, and Sigmoid are the activation functions from which to choose.

Iteration limit — Provide the full number of training iterations.

Regularization strength (Lambda): Provide the ridge (L2) regularization penalty term.

Standardize data: Indicate if you want the numerical predictors to be standardized. Standardizing predictors with very disparate scales can enhance the fit. Standardizing the data is strongly recommended.

Where:

• Input: This layer matches the data from the predictor.
• FC: First fully connected layer.
• ReLU: The first fully connected layer has been applied by activation function.
• Softmax: fitcnet function for Binary and multiclass classification uses the last completely connected layer to apply this activation function. The function accepts an input of type *x*_*i*_ and outputs the following, where *k* is the response variable's class count.:
  (1) $$f\left(x_{i}\right)=\frac{\exp\left(x_{i}\right)}{\sum_{j=1}^{k}\exp\left(x_{i}\right)}$$

The predicted classification scores of results correspond (or posterior probabilities).

Output: The predicted class labels of this layer correspond.

#### Classification scores

The scores of classifications for a neural network classifier are calculated using the softmax activation function that follows the final fully connected layer in the network [33]. The posterior probabilities of scores correspond, the posterior probability that an observation x is of class k is

(2) $$\begin{array}{ccc} \hat{P}\left(k|x\right) & = & \frac{P\left(x|k\right)P\left(k\right)}{\sum_{j=1}^{k}P\left(x|j\right)P\left(j\right)}\\ & = & \frac{\text{exp}\left(a_{K}\left(x_{i}\right)\right)}{\sum_{j=1}^{k}\exp\left(a_{j}\left(x_{i}\right)\right)} \end{array}$$

Where:

• *P*(*x*|*k*) is the conditional probability of *x* given class *k*.
• *P*(*k*) is the prior probability for class *k*.
• *k* is the number of classes in the response variable.
• *ak*(*x*) is the *k* output from the final fully connected layer for observation *x*.

Select a validation technique to assess the fitted models' prediction accuracy. By comparing the model's performance on fresh data to the training set, validation helps determine which model performs best. Overfitting is prevented by validation. Prior to training any models, decide on a validation technique so that you may use the same validation scheme to compare all of the models in your session.

Assume that no data is reserved for testing, which is true by default [34].

• Cross-Validation: To partition the data set by select a number of divisions (or folds). Where k-fold is modified, then the app:
  1. splits the information into k folds or distinct sets.
  2. For each validation fold:
    a. To use training-fold observations by trains a model.
    b. Evaluates the performance of the model with validation-fold data.
  3. Estimates the average validation error over all folds.

The predicted accuracy of the final model that was trained using all the data is fairly estimated using this technique. It is advised for small data sets since it needs several fits yet effectively uses all of the data.

• Holdout Validation: Decide which proportion of the data will serve as the validation set. Using the training set, the software trains a model, then uses the validation set to evaluate its performance. Holdout Validation is only advised for big data sets because the validation model is only based on a subset of the data. The entire data set is used to train the finished model.
• Resubstitution Validation: To create a validation set, choose a percentage of the data. A model is trained on the training set using the program, and its performance is evaluated using the validation set. Only large data sets are advised for Holdout Validation since the model used for validation is based on a small fraction of the data. All of the data is used to train the final model.

## Result and discussion

Several datasets, including Messidor, IDRiD, and others, are made up of high-quality images that were taken in regulated, unusual settings (i.e. similar hardware and environmental conditions across captures). Because the images may not be exactly equivalent and the technology and environment may differ, one may claim that algorithms trained on such datasets will perform badly in normal actual settings. However, even though the Kaggle EyePACS and APTOS datasets deal with these problems and are quite like real-world scenarios, because they are made up of images taken from a range of camera models under a variety of non-typical conditions, the noise resulting from those variations makes it extremely challenging for the algorithms to carry out the analysis in an accurate and efficient manner.

**Fig. 7:**
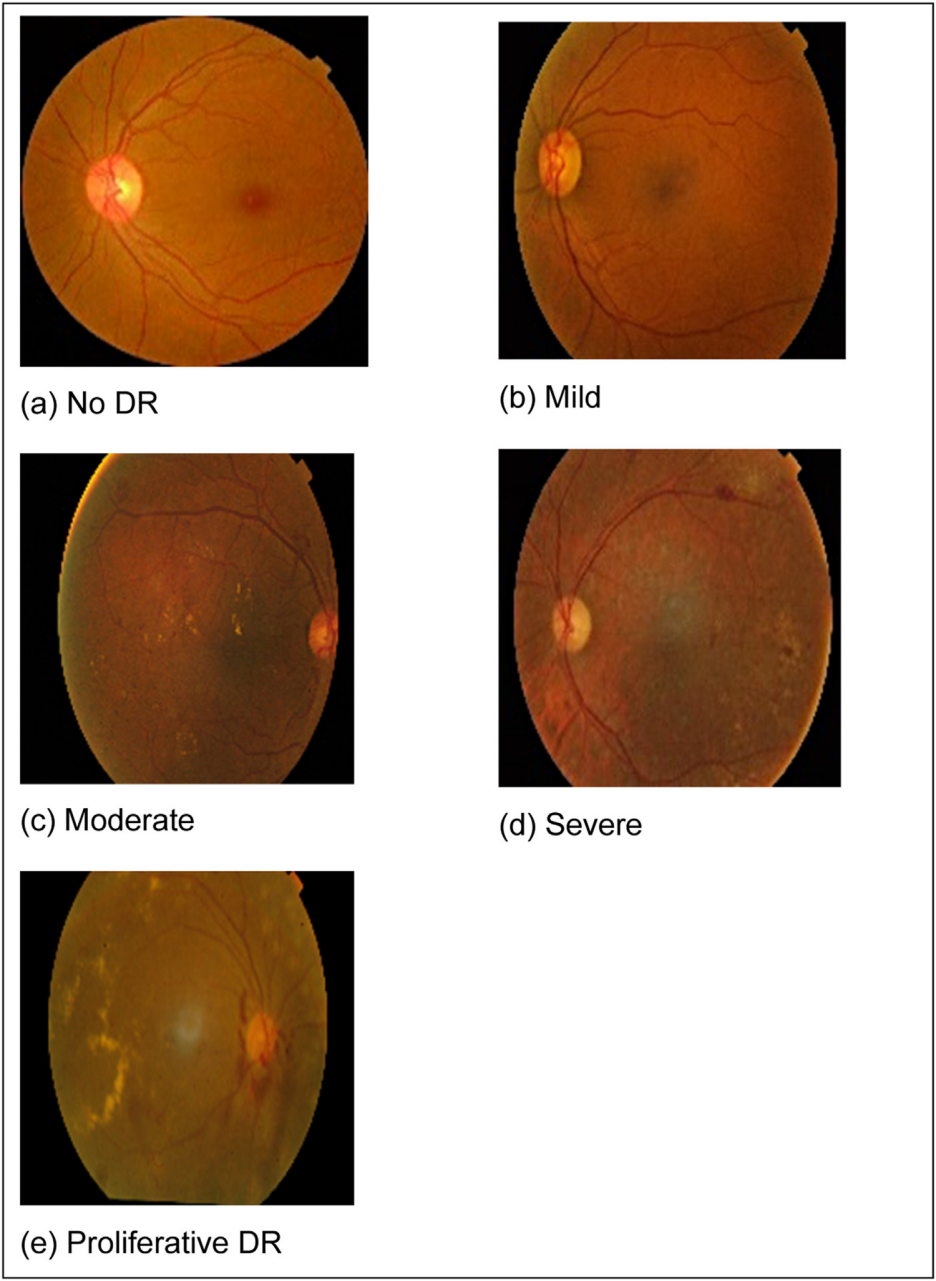
Different Classes of Diabetic Retinopathy.

Collection of retinal images into dataset. EyePACS contributed the 3662 retinal images that make up the input dataset used in this work, which was obtained via Kaggle. Additionally, it separates into 550 images for testing and 3112 images for training. Different noise levels and quality settings were used to apprehend the images in this collection. The severity rating ranging from 0 to 4 in the dataset, and it indicates the level of DR present in image. A class of 0 indicates a normal image with no signs, class 1 denotes mild, class 2 indicates moderate, class 3 reveals severe, class 4 signifies non proliferative DR [10], as shown in Fig. 7.

The number of images in each stage in the dataset for different stages of retinal images are mentioned in Table 2.

**Table 2:** Different Stages for Identification of Image.

#	Stage	Images Identification
0	Non (Normal)	1805
1	First (Mild)	370
2	Second (Moderate)	999
3	Third (Severe)	193
4	Fourth (Pro-DR)	295

**Fig. 8:**
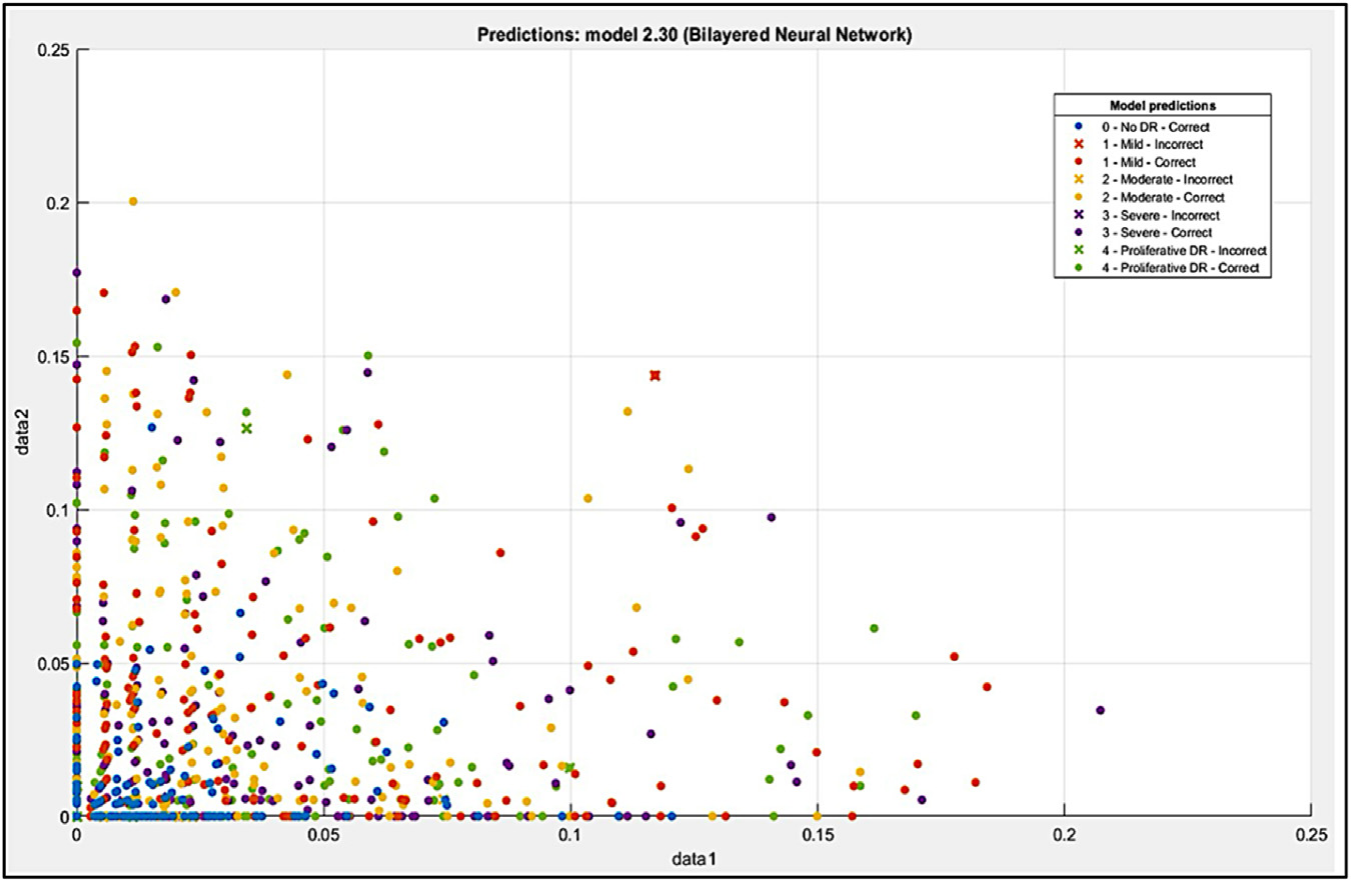
Extracted Features Scatter.

The vSLAM method has been selected for feature extraction, it may create a bag of visual words—roughly 500 visual characteristics simultaneous localization for each image—for use in loop closure detection, image retrieval, and image category classification in visual simultaneous localization and mapping samples., as shown in Fig. 8.

Nonetheless, by considering those low-quality images that accurately depict the data, strong algorithms that are useful in clinical settings may be created. In the paper, MATLAB Platform environment is used for the examination. It allows scripting and executing code for function of classification model. 80 GB of a disk storage memory has been set aside for this work. In the end, it is split into two sets: 15% is used for testing and 85% is used for training. The dataset collected of completely 3662 images, in that 3112 images is applied for training and 550 images is used to testing. The obtained results using Three Validation cross, holdout and Resubstitution, the good result have been got under Resubstitution validation which was 98.5% shown in Table 3.

**Table 3:** Final Validation Result.

Model Type	Accuracy % (Validation)		
	Cross	Holdout	Resubstitution
Tree	41.64706	42.92453	87.64706
Discriminant	38.35294	35.84906	96.58824
Efficient Logistic Regression	42.82353	42.92453	46.82353
Efficient Linear SVM	50	50	64.23529
Naive Bayes	NaN	44.33962	86.82353
SVM	53.17647	51.41509	97.29412
KNN	47.17647	47.16981	96.33124
Ensemble	49.76471	47.64151	97.29412
Neural Network	47.05882	49.5283	98.51176
Kernel	NaN	51.41509	89.17647

Above result which this research has been reached after trained and tested in different classification mode. The error is known as the resubstitution error in Resubstitution Validation if all the data is utilized to train the model and the error rate is assessed using result vs. actual value from the same training data set. The resubstitution validation methodology is the name given to this method. The full dataset is used to train the model in the resubstitution validation procedure. This method compares the actual values from the same training dataset with the expected outcomes to assess the model's performance. The resubstitution error rate, which shows how well the model predicts the data it was trained on, is obtained from this comparison. With this approach, the correctness of the model is evaluated on the training data alone, without the need for an additional test set.

When using the resubstitution validation approach, all of your data are used as training data. Next, you do a comparison between the actual value from the training data set and the error rate of the output of the machine learning model. This is a simple strategy that will assist you in rapidly identifying any gaps in your data.

**Fig. 9:**
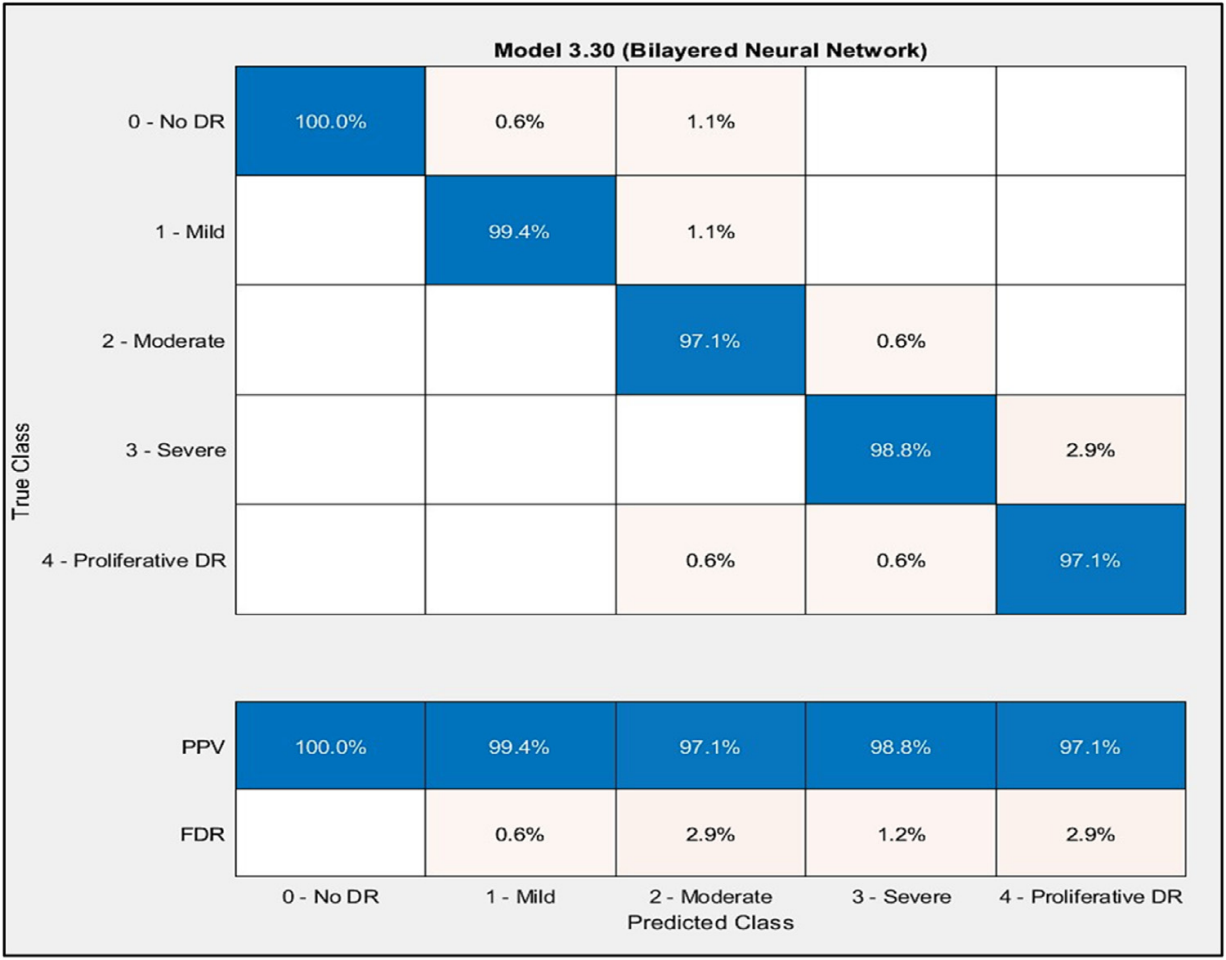
Confusion Matrix of Bilayered Neural Network.

Bilayered Neural Network model get the highest accuracy result, in that reason have been chosen in this paper research, beside that it was had average number of layers in all type of Neural Network, that it be to categories stage of diabetic retinopathy for each No DR, Mild, Moderate, Severe and Proliferative DR fundus image. To predicate each of class, the focus proposed work are on No DR class that be in every test be on highest precision, because others are the person have Diabetic, here the most important is to know that person in normal case or not, and then to classify the stage of diabetic. Predicated class result for each stage of Diabetic Retinopathy under Bilayered Neural Network model with PPV and FDR as showing in Fig. 9.

**Fig. 10:**
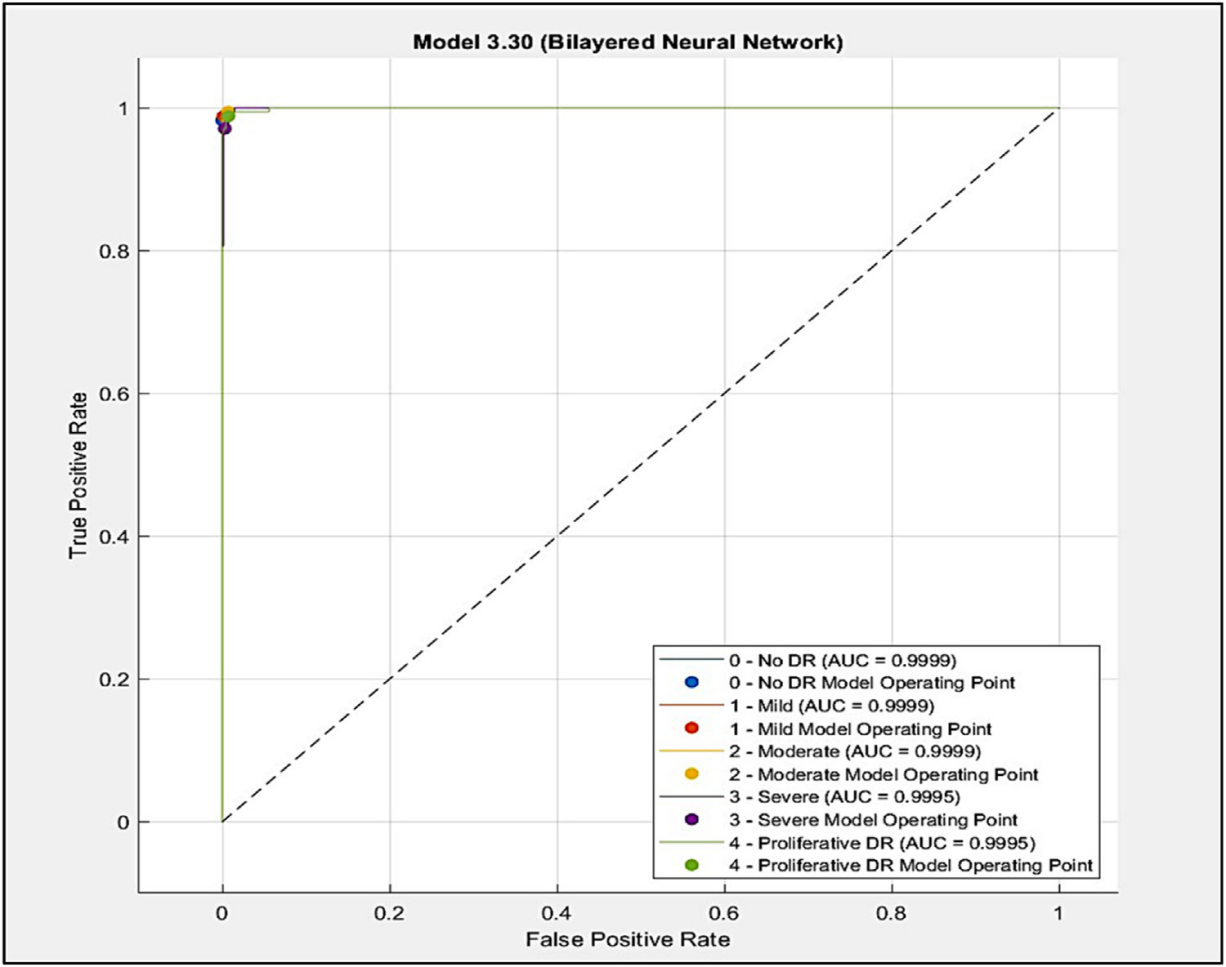
AUC of Bilayered Neural Network.

The DREAM system achieves 100% No DR with 0.9999 AUC, 99.4% mild with 0.9999 AUC, 97.1% moderate with 0.9999 AUC, 98.8% severe with 0.9995 AUC, and 97.1% proliferative DR with 0.9995 AUC, under Bilayered Neural Network model as showing in Fig. 10.

Comparing the current proposed work with other diabetic retinopathy (DR) detection research involves using different datasets and classifiers, as mentioned above in the previous discussion, have been illustrated in the below comparison Table 4. The classifier, dataset, and feature extraction are compared with the previous models compared with the proposed research.

**Table 4:** Comparison of Proposed Work with Previous Model.

#	Authors	Dataset	Classifier	Feature Extraction	Experimental Results Accuracy
1	Raj et al. [4]	Kaggle Dataset (IDRiD)	CNN	Microaneurysms, Blood Vessels	95.41%
2	Kamblea and Kokate [5]	DIARETDB1 Dataset	RBF Neural Network	Microaneurysms, Exudates, blood vessels	89.4%
3	Alyoubi et al. [6]	Aptos 2019 Dataset	CNN	DR Lesions Found	89%
4	Kolla and Venugopal [17]	Messidor Dataset	SVM	Blood vessels, microaneurysms and hard exudates	94%
5	Yu et al. [21]	NVD Image Dataset	SVM	Optic Disc Blood Vessels	95.23%
6	Raja Sarobin et al. [22],	Kaggle Dataset (IDRiD)	DenseNet	DR lesions found	95.22%
7	Agurto et al. [24],	Messidor Dataset	KNN	Exudates	97.7%
8	Zubair et al. [25]	STARE Dataset	DCNN	Microaneurysms, Hemorrhages, Exudates	95.33%
9	Pradeep et al. [10]	Kaggle Dataset (IDRiD)	CNN	Optic Disc, Fovea, Hemorrhages, Microaneurysms	97.7%
10	Proposed Model	Kaggle Dataset (IDRiD)	Bilayered Neural Network	Optic Disc, Fovea, Hemorrhages, Microaneurysms	98.5%

The suggested study uses several validation modes and machine learning and classification model approaches to detect DR. Kaggle dataset (IDRiD) have been used in this suggested system for training and testing the images with Bilayered Neural Network (BNN). When BNN approaches are compared for the identification of diabetic retinopathy, it is discovered that these methods have a high accuracy rate of 98.5%. However, when compared to other models, the BNN outperforms in terms of accuracy overall performance. As a result, we may infer that BNN is a more appropriate and successful approach for predicting diabetic retinopathy from retinal fundus images.

## CRediT authorship contribution statement

**Herman Khalid Omer:** Methodology, Software, Writing – original draft.

## Declaration of competing interest

The authors declare that they have no known competing financial interests or personal relationships that could have appeared to influence the work reported in this paper.

## Data Availability

No data was used for the research described in the article.
